# Displacement Following Minimally Invasive vs Open Lateralizing Calcaneal Osteotomy: A Direct Comparison in a Cadaveric Model

**DOI:** 10.1177/24730114261463248

**Published:** 2026-07-15

**Authors:** Hirbod Abootalebi, William Mayer, Erin Bigney, Oreoluwa Alugo, Rowan Urquhart, Jacob Matz

**Affiliations:** 1 Dalhousie Medicine New Brunswick, Saint John, NB, Canada; 2 Canada East Foot & Ankle, Saint John, NB, Canada; 3 Faculty of Medicine, Department of Medical Neuroscience, Halifax, NS, Canada; 4 Horizon Health Network, Saint John Regional Hospital, Saint John, NB, Canada; 5 Canada East Spine Centre, Saint John, NB, Canada; 6 Saint John Orthopaedics, Saint John, NB, Canada

**Keywords:** calcaneal osteotomy, cavovarus foot, hindfoot varus, minimally invasive surgery

## Abstract

**Background:**

Minimally invasive (MIS) calcaneal osteotomy is increasingly used to decrease soft tissue morbidity, but whether MIS lateral displacement calcaneal osteotomy (LDCO) can achieve similar translation to the open calcaneal osteotomy technique remains unclear.

**Methods:**

In a paired cadaveric study, 16 feet from 8 donors were block-randomized so that each pair received 1 open LDCO and 1 MIS LDCO performed by a fellowship-trained foot and ankle surgeon. Lateral translation of the tuberosity was measured directly via a medial approach using a metric ruler by 2 independent observers; Wilcoxon signed-rank test (α = 0.05) compared the 2 techniques. We operationally defined ≥5 mm as a clinically meaningful threshold.

**Results:**

Median lateralization was 5.0 mm (range 3.0-8.0) for MIS and 7.5 mm (range 4.0-9.5) for open. The Hodges-Lehmann median difference was 2.41 mm (95% CI: 1.25-4.00 mm), with no statistically significant difference detectable with the numbers available between techniques (Z = −1.47, *P* = .14). Using a clinically relevant ≥5 mm threshold, 4 of 8 MIS specimens fell below this level compared with 2 of 8 open specimens.

**Conclusion:**

Both open and MIS LDCO were associated with similar lateral translation of the calcaneal tuberosity, with no significant difference detectable with the numbers available, though MIS was less consistent in reaching ≥5 mm of lateralization. Either approach may be used for mild-to-moderate hindfoot varus, with technique selection guided by soft tissue and infection risk considerations.

**Clinical Relevance:**

For mild to moderate hindfoot varus correction, MIS LDCO provides deformity correction comparable to the open approach while potentially reducing soft tissue morbidity. Surgical approach selection should consider patient-specific risk factors.

## Graphical abstract

**Visual Abstract:**
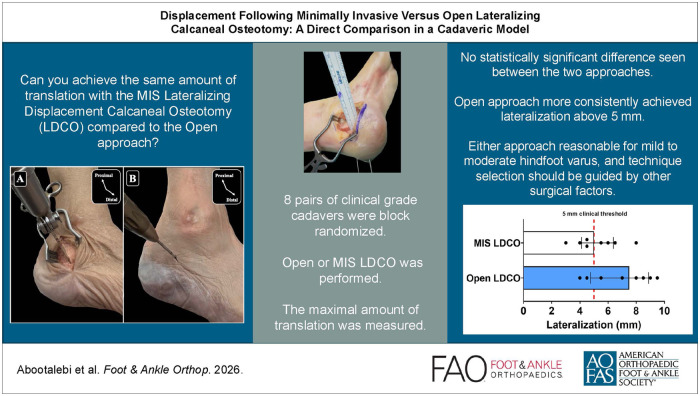

## Introduction

Cavovarus foot deformity is a multifactorial condition characterized by a varus hindfoot, high medial longitudinal arch, and forefoot malalignment, usually caused by an interplay of bony abnormalities, soft tissue contractures, and neuromuscular conditions such as Charcot-Marie-Tooth disease.^1-3^ Previous work has shown that the pathoanatomy leads to altered load distribution across the foot, leading to compensatory changes in the foot that further exacerbate the deformity.^4,5^ Over time, structural abnormalities lead to lateral column overload, which can contribute to pain, instability, and the development of ankle arthritis.^3,6^

The surgical correction of cavovarus feet frequently requires multiple procedures, addressing both the bony and soft tissue aspects of the deformity.^2^ The varus deformity of the hindfoot is frequently addressed in a joint-sparing fashion using a calcaneal osteotomy. The most common procedure, lateral displacement calcaneal osteotomy (LDCO), is designed to achieve lateral translation of the calcaneal tuberosity to correct the hindfoot varus component of the deformity. A number of other calcaneal osteotomies exist, including lateral closing wedge (Dwyer osteotomy), lateral closing wedge with lateral translation (modified Dwyer), Z-type osteotomy (Malerba), among others.^7,8^

Traditionally, LDCO is performed through an open approach that exposes the lateral wall of the calcaneus, allowing direct visualization and precise control of the osteotomy. Complications that have been described with open approaches for calcaneal osteotomies include wound healing delays (5%-10%)^9,10^ and sural nerve injuries (7%-25%).^11-14^ Cavovarus reconstructions frequently entail multiple lateral sided procedures in addition to a calcaneal osteotomy, such as peroneal tendon and lateral ankle ligament repairs, making wound healing complications and skin bridge necrosis a pertinent risk.

Minimally invasive (MIS) calcaneal osteotomies may reduce the risk of soft tissue complications (<1%-3%), potentially making these a valuable adjunct in cavovarus reconstructions.^15,16^ Although the MIS approach may be less prone to soft tissue healing complications, the amount of lateral translation achievable with an open vs MIS osteotomy has not been quantified. This represents an important gap in the literature since the extent of lateral translation of the weightbearing surface of the calcaneus is directly associated with the effectiveness of deformity correction.^7^ The present cadaveric investigation compares the amount of translation achievable after open LDCO vs an MIS LDCO in paired cadaveric specimens. Our hypothesis was that the open approach, through additional soft tissue release, will allow for greater translation than the MIS approach. Establishing the extent of translation achievable with each technique is important to facilitate preoperative planning and aid intraoperative decision making.

## Methods

### Specimens

Sixteen human cadaveric specimens from 8 body donors, spanning from mid-tibia to the tip of the toes, were used. These comprised 2 male and 14 female specimens of Caucasian ethnicity. Each specimen was radiographed to ensure no signs of musculoskeletal abnormalities, previous fracture, or surgery. The mean age of the specimens was 86.9 years (range: 76-96 years). The specimens were prepared with the Sandeski Clinical Grade Cadaver technique (previously known as the Halifax Clinical Cadaver Preparation) and stored at 4 °C.^17^ Specimens were block randomized for sidedness, with one foot in each pair assigned to either an open osteotomy or a minimally invasive LDCO. The contralateral foot received the alternative procedure. In both groups, a fellowship-trained foot & ankle orthopaedic surgeon performed all surgeries. This study was approved by the Horizon Health Network Research Ethics Board under RS no. 2025-3481.

### Open Osteotomy Technique

**Figure 1.**
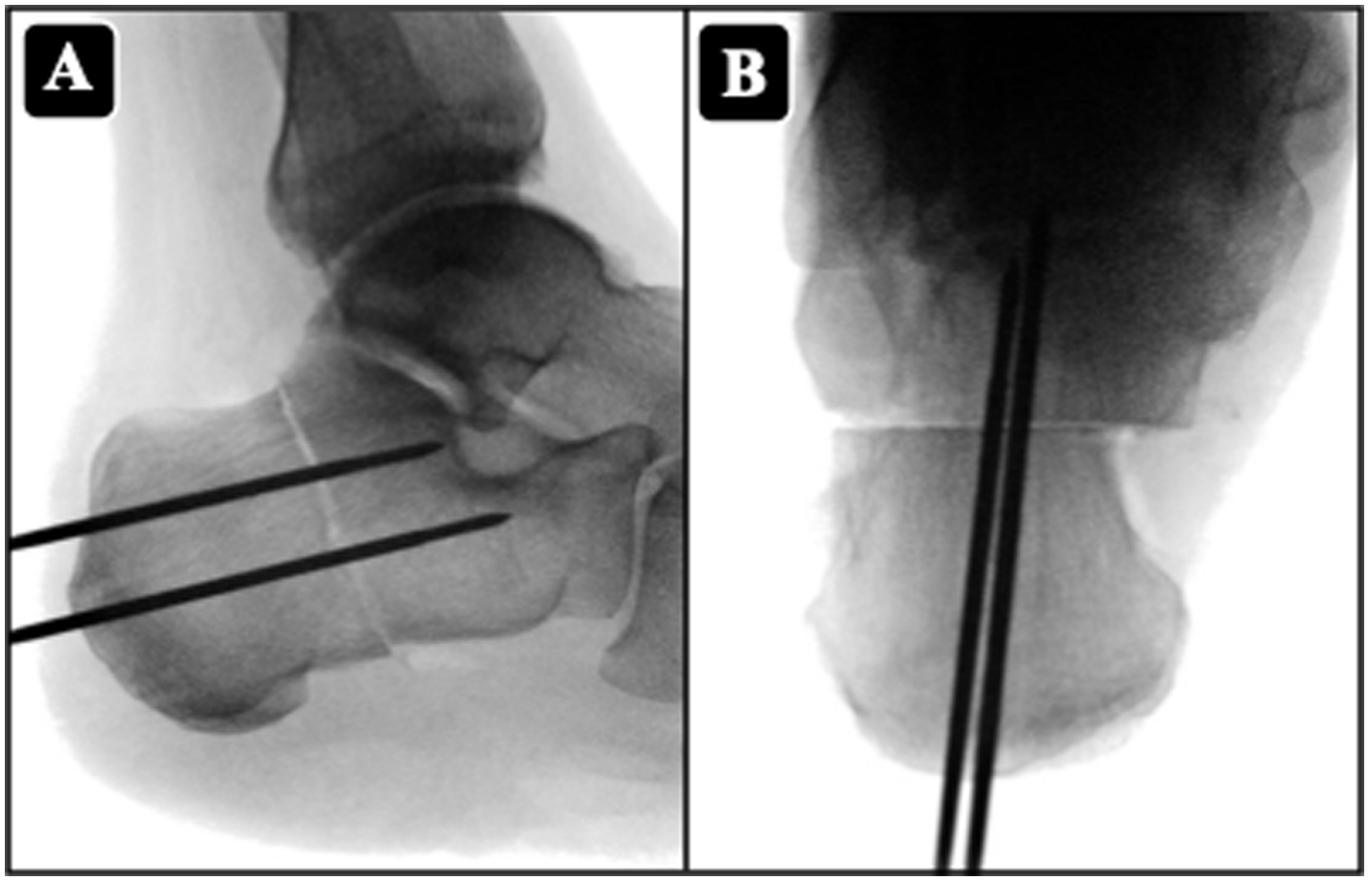
(A) Lateral and (B) axial fluoroscopic images post lateral translation using the open osteotomy approach.

Fluoroscopy was used to identify the position for the osteotomy as per the safe zone outlined by Talusan et al.^18^ An incision was made along this line and the sural nerve was protected. The periosteum was sharply elevated. Two Kirschner (K)-wires were inserted to demarcate the position of the osteotomy and ensure that the osteotomy is perpendicular to the long axis of the calcaneus. This was checked with fluoroscopy on a Harris axial view. A micro-sagittal saw (20 × 40 mm, Hall), with saw blade thickness of 0.4 mm, was used to complete the osteotomy. A laminar spreader was inserted into the osteotomy, then distracted, and a medial soft tissue release was carefully carried out. K-wires (2.5 mm) were inserted into the tuberosity and used to translate the tuberosity maximally laterally and then advanced into the calcaneus to secure in place (Figure 1). Although the force applied was not measured, this method was followed consistently across all specimens and reflects the technique used in clinical practice. A Harris axial view fluoroscopic image was taken to ensure the K-wires were in the appropriate trajectory to secure the osteotomy.

### MIS Lateralizing Osteotomy Technique

**Figure 2.**
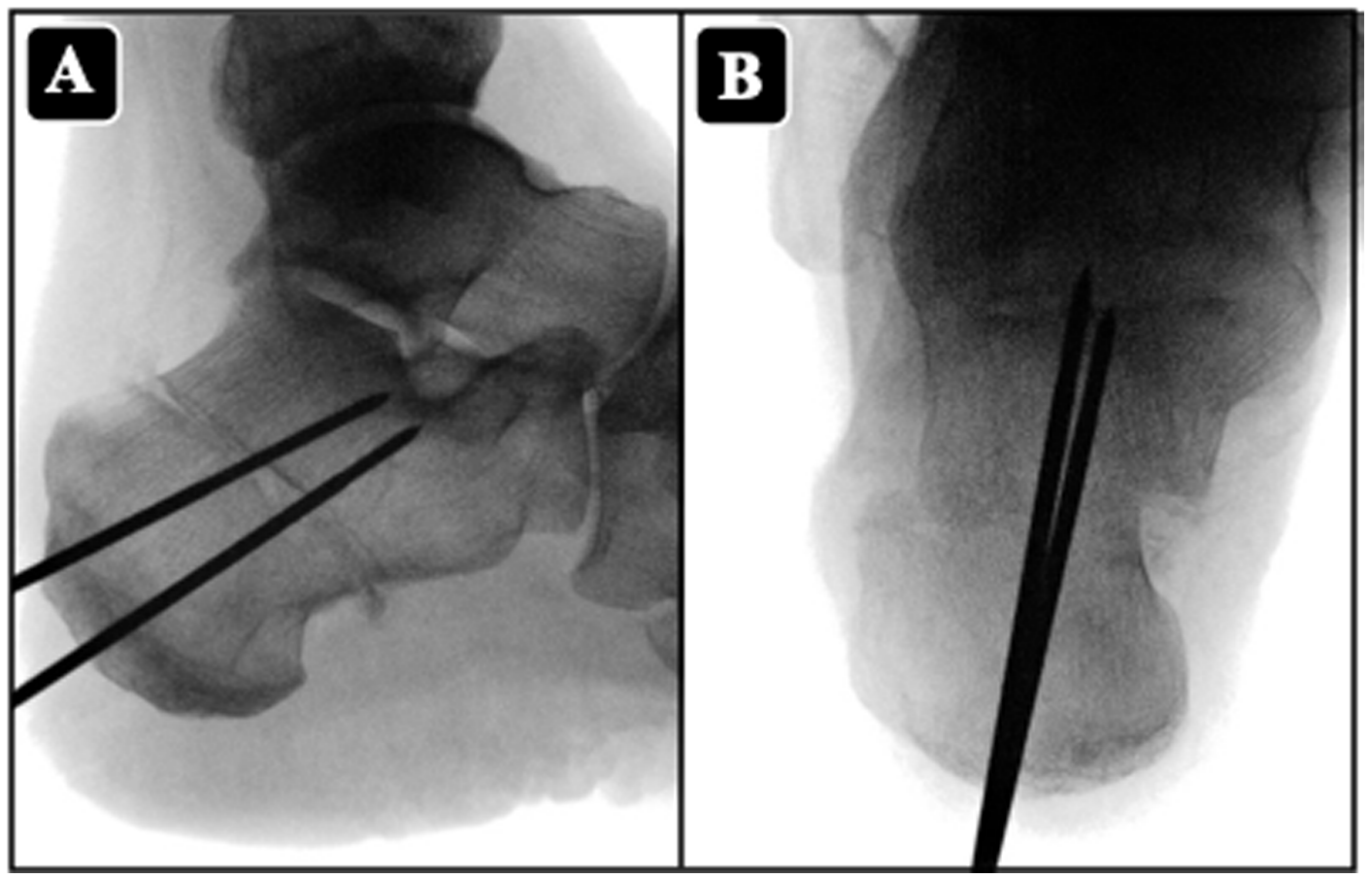
(A) Lateral and (B) axial fluoroscopic images post lateral translation using the MIS osteotomy approach.

Under fluoroscopic guidance, the osteotomy site was identified anterior to the origins of the plantar fascia and the Achilles tendon, following the safe-zone parameters described by Talusan et al.^18^ A 5-mm longitudinal incision was made over the mid-calcaneus, and blunt dissection was carried out to expose the lateral cortical surface of the calcaneus. A high-speed, low-torque Shannon burr (2 × 19.5 mm) was then used to perform the osteotomy using a “four quadrants” technique.^19^ The burr was first centered on the calcaneal tuberosity and its perpendicular alignment verified with an axial fluoroscopic image. Sequentially, the lateral quadrants were cut followed by the medial quadrants until the tuberosity became mobile. Two 2.5-mm K-wires were subsequently inserted into the posterior aspect of the tuberosity; these served both as joysticks to achieve maximal lateral translation and as fixation devices to secure the displaced segment (Figure 2). A Harris axial view fluoroscopic image was taken to ensure the K-wires are in the appropriate trajectory to secure the osteotomy.

### Measurements and Statistical Analysis

**Figure 3.**
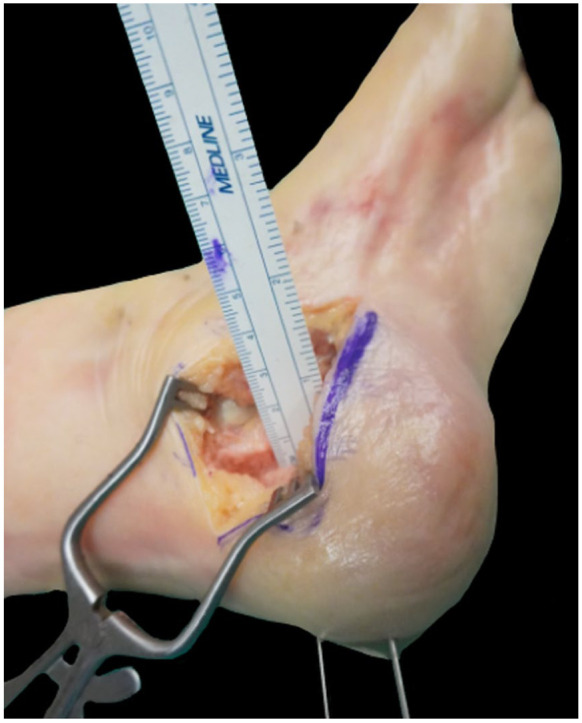
Clinical measurement using a ruler placed through a medial approach following an open osteotomy.

In both groups, clinical measurements were obtained to quantify the amount of lateral displacement of the calcaneal tuberosity. The medial side of the calcaneus allowed accurate measurements without distortion of the cortex from the osteotomy procedure itself. Hence, through a separate medial approach, the medial wall of the calcaneus and the translated tuberosity were exposed. This permitted a direct measurement of the maximal displacement of the osteotomy in the center of the calcaneus using a metric ruler (Figure 3).

In addition to the clinical assessment, we considered measuring the tuberosity translation using the axial fluoroscopic view. However, because of the obliquity of the calcaneal surface, and the nonlinear trajectory of the osteotomy, obtaining a perfectly parallel fluoroscopic view to the osteotomy was not feasible, particularly in the MIS group. Hence, radiographic measurements were inherently unreliable and were not utilized.

All clinical measurements were taken by 2 investigators independently, and the median was calculated. Differences in lateralization (mm) between the 2 procedures were compared using the Wilcoxon signed-rank test due to the small sample size and non-normal distribution. Statistical significance was set at α = 0.05. The Hodges-Lehmann estimator was used to calculate the median difference and its 95% CI. Given that no minimum has been established and previous papers have reported displacement from 5 to 10 mm of lateralization, we defined ≥5 mm as a conservative, clinically meaningful threshold.^7,16,20^

## Results

Lateralization was lower for MIS (median = 5.00 mm, range 3.00-8.00) compared with open (median = 7.5 mm, range 4.00-9.50) (Table 1). The median difference in lateralization was 2.41 mm (95% CI: 1.25-4.00 mm), estimated using the Hodges-Lehmann method. There is no statistically significant difference detectable with the numbers available between the groups (Z = −1.47, *P* = .14).

**Table 1.** Comparison of the lateral translation achieved with open versus minimally invasive lateralizing calcaneal osteotomy.^a^

Cadaver	Open (side; mm)	MIS (side; mm)
1	L; 8.0	R; 3.0
2	L; 4.5	R; 6.0
3	R; 4.0	L; 5.5
4	R; 5.5	L; 4.5
5	R; 9.0	L; 8.0
6	L; 7.0	R; 4.5
7	L; 9.5	R; 6.5
8	R; 8.5	L; 4.0
Median (range)	7.5 (4.0-9.5)	5.0 (3.0-8.0)

Abbreviations: L. left; MIS, minimally invasive surgery; R, right.

a Wilcoxon signed-rank: Z = −1.47, *P* = .14. Hodges-Lehmann estimator (Open − MIS): 2.41 mm (95% CI: 1.25-4.00 mm).

**Figure 4.**
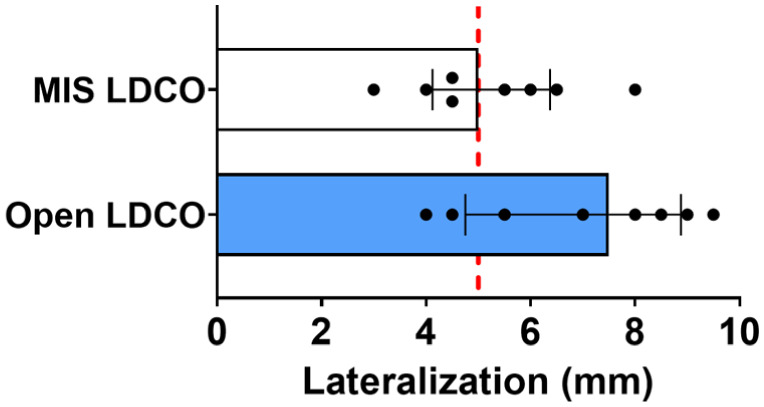
Median lateralization achieved with MIS and Open LDCO techniques. Error bars represent the interquartile range; individual dots reflect specimen-level measurements. The dashed red line denotes the predefined 5-mm clinical threshold. LDCO, lateral displacement calcaneal osteotomy; MIS, minimally invasive surgery.

When considering the threshold of 5 mm, 4 of 8 feet in the MIS group failed to meet that threshold compared with 2 of 8 in the open group (Figure 4).

## Discussion

When comparing open vs MIS techniques for LDCO, direct clinical measurements did not demonstrate a statistically significant difference in the amount of lateral translation achieved. However, when results were interpreted relative to a clinically relevant benchmark, a greater proportion of specimens failed to meet the minimum threshold in the MIS cohort (50%) compared to the open cohort (25%). Given that a similar extent of deformity correction can be obtained by open vs MIS LDCO technique, other factors such as soft tissue quality, and co-existing incisions, should be considered when deciding between open vs MIS LDCO.

Salameh et al^21^ recently compared open vs saw-based MIS techniques for medial displacement calcaneal osteotomy in a cadaveric study and reported no significant difference between techniques (manual measurement: 7.9 mm MIS vs 8.7 mm open). Several methodologic and biomechanical differences may explain the different findings between our studies. First, lateral translation may be inherently more constrained than medial translation because of the presence of medial soft tissue structures including the flexor retinaculum, tarsal tunnel contents, abductor hallucis and the plantar fascia.^22,23^ This anatomic consideration may explain why medial displacement calcaneal osteotomy achieved overall greater translation averages compared with our LDCO results. Second, in our open technique, a laminar spreader was introduced into the osteotomy to help overcome the constraints imposed by soft tissues on lateral translation, whereas our MIS technique relied on the removal of bone that facilitates the tuberosity mobility. Salameh et al^21^ performed medial translation, which is less constrained by soft tissues, potentially explaining why their laminar spreader use (open) or absence (MIS) had minimal impact on translation distances. Third, our measurement methodology differed. We used direct clinical measurements from the medial cortex, which provided accurate assessment without osteotomy-related distortion. We avoided radiographic measurements because obtaining a perfectly tangential fluoroscopic view relative to the osteotomy plane was not feasible, particularly with MIS approach, and any angulation led to measurement inaccuracy. Salameh et al^21^ used both radiographic and manual measurements from a lateral approach. This methodological difference may partially explain the discordance between our findings.

Several surgical techniques can improve the ability to translate a calcaneal osteotomy. With open osteotomy, it is important to obtain a Harris axial view with a templating wire to confirm that the trajectory of the osteotomy is perpendicular to the long axis of the calcaneus. Osteotomies that inadvertently lengthen the calcaneus make tuberosity translation substantially more constrained. Following completion of the osteotomy, translation is enhanced by the insertion of a laminar spreader into the osteotomy, distraction, and careful soft tissue release on the medial side. The saw blade thickness for the open osteotomy was 0.4 mm compared with 2 mm for the MIS burr. Although this increased kerf is thought to facilitate tuberosity translation in the MIS technique, our results did not support this. It is possible that increasing the kerf of either instrument would further reduce soft tissue tension and allow for greater translation, although this was not specifically evaluated in this study.^24^ The insertion of robust joysticks into the calcaneal tuberosity can facilitate maximal lateral translation with MIS LDCO. For both open and MIS technique, staying anterior to the origin of the plantar fascia and the insertion of the Achilles tendon is critical to avoid significant soft tissue constraints to translation.^24^

Comparative data on the ability of open and MIS techniques to translate an LDCO is scarce. Kendal et al^16^ conducted a retrospective cohort study evaluating the effectiveness and complication rates of open vs minimally invasive (MIS) calcaneal osteotomies for both planus and cavus deformities. Of the 81 patients included, 14 (17.3%) underwent a lateral displacement calcaneal osteotomy (LDCO), with 10 in the open group and 4 in the MIS group. The study reported comparable effectiveness between the 2 techniques, with mean translations of 9.4 mm (SD 1.16) for the open approach and 10.4 mm (SD 1.06) for MIS.^16^ Other studies compared the various types of open lateralizing calcaneal osteotomies. An et al^7^ used a 3D-printed model to assess the effects of various open osteotomy techniques on the translation of the weight bearing surface of the calcaneus, finding that a closing wedge osteotomy in combination with lateral translation (modified Dwyer) and internal rotation was the most powerful technique. Similar results were obtained in a 3D modeling study, confirming that the greatest shift is obtainable with a modified Dwyer.^25^ Hence, although we found that open and MIS LDCO have similar corrective power, suitable for mild to moderate deformity, in cases with severe deformity where maximal lateralization of the weightbearing surface is required, the combination of open LDCO with a closing wedge osteotomy (modified Dwyer) is likely preferable.

**Figure 5.**
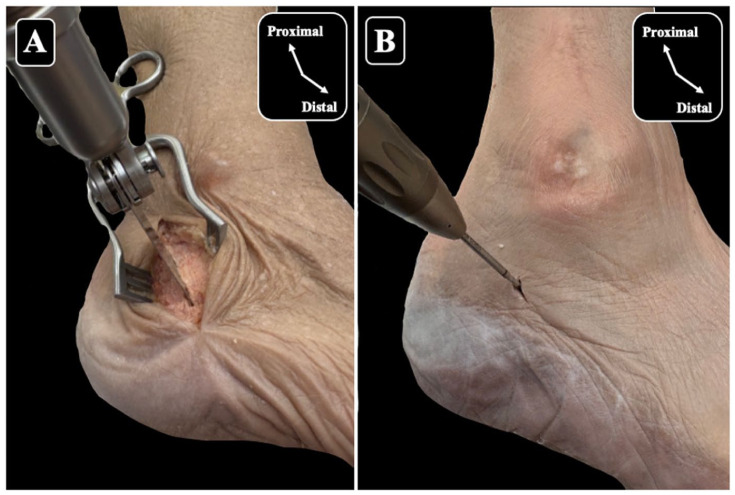
Lateral intraoperative images of the incision site difference between open (A) and MIS (B) approaches, with corresponding use of the oscillating saw (A) and high-speed burr (B) to illustrate the surgical techniques.

Clinical studies examining calcaneal displacement osteotomies have shown lower rates of wound complications and infections with MIS techniques.^15,16,26,27^ Kendal et al^16^ found that wound complications were nearly 5 times more frequent in the open group compared with MIS (28% vs 6.5%), with similar trends for infections (20% vs 3%), although the majority of their cohort underwent medializing procedures for flatfoot reconstruction. Gutteck et al^27^ found comparable clinical outcome scores but a substantial reduction in wound healing complications with the percutaneous technique. deMeireles et al^15^ reported an overall very low rate of complications (3.4%) with the MIS calcaneal osteotomy technique, consisting of transient neuritis (1.7%), prolonged drainage (0.8%), and non-union (0.8%). Coleman et al^26^ were able to identify populations that may be at increased risk for osteotomy and wound healing complications with minimally invasive and percutaneous techniques, consisting of patients with higher ASA classification, current tobacco use, higher BMI, and those undergoing more extensive reconstructions. These lower complication rates are largely attributed to the smaller incision size and decreased soft tissue stripping of the MIS approach (Figure 5), a particularly valuable advantage in cavovarus reconstructions where multiple lateral incisions may be required. Although a 5-mm threshold was defined in this study and 50% of MIS specimens fell below this correction, its lower risk of infection and soft tissue complications may be more clinically important.

Calcaneal osteotomies can lead to neurologic complications. On the lateral side, the sural nerve and its branches are susceptible to injury.^28^ In a series of 76 open calcaneal osteotomies, González-Martín et al^28^ found a 26% incidence of sural nerve injury. Coleman et al^26^ reported on 189 percutaneous and minimally invasive calcaneal osteotomies, finding an overall 8% rate of sural nerve deficits. Other authors reported lower rates associated with the MIS technique, but the risk still needs to be recognized.^9,10,15,16^ On the medial side, the tibial nerve and its branches are at risk both during the osteotomy as well as following the tuberosity shift. This risk is particularly pertinent for lateralizing osteotomies that carry a significant rate of tibial nerve deficits and tarsal tunnel syndrome.^20,28,29^ Biomechanical and imaging studies demonstrated that lateralizing the calcaneal tuberosity reduces the volume of the tarsal tunnel and increases intracanal pressures, especially with greater degrees of translation.^7,22,30^ In the subset of 28 feet that underwent a pure translational lateralizing slide, VanValkenburg et al^20^ documented tibial nerve sensory deficits in 7 cases (25%). González-Martín et al^28^ found neurological injuries in 16 of 42 lateral slides (38%) and noted tibial nerve or branch involvement in 21% of cases. Stødle et al^29^ documented tibial nerve palsy in 3 of 18 lateral slides (17%). Whether these deficits are due to the osteotomy itself or the result of the tuberosity shift is unclear. Some authors recommend prophylactic release of the flexor retinaculum when planning larger lateral shifts to mitigate the risk of tibial nerve compression.^30,31^ More recently, it has also been shown that MIS lateralizing osteotomy using a burr instead of a saw leads to a significantly smaller rise in tarsal tunnel pressures, suggesting a possible way to lower the risk of postoperative nerve symptoms.^32^ In summary, whether open or MIS technique is used, lateralizing calcaneal osteotomies can lead to neurologic complications, highlighting the need for meticulous surgical technique, careful soft tissue handling, and thorough patient counselling.

This study has several limitations. First, our sample size is limited and underpowered to detect small but potentially clinically meaningful differences; however, this is similar to other cadaveric investigations.^21^ Second, the cadaver specimens are from elderly donors (mean age = 86.9 years) and were exclusively of Caucasian ethnicity, which may not fully capture the variability seen across the general population.^33^ Most importantly, these specimens did not have cavovarus deformities, which could alter soft tissue tension, potentially affecting the amount of translation achieved during surgery.

## Conclusion

This cadaveric study directly compared open and minimally invasive LDCO and found that although no statistically significant difference was detected between MIS and open LDCO in the ability to produce lateral translation of the calcaneal tuberosity, more MIS specimens fell below the ≥5-mm threshold, suggesting it may be less consistent in reaching clinically meaningful lateralization. Ultimately, both the open and minimally invasive approaches offer similar corrective power detectable with the numbers available for mild to moderate hindfoot varus deformity, and the approach choice should be dictated by the perceived risk to the soft tissues.

## Supplemental Material

sj-pdf-1-fao-10.1177_24730114261463248 – Supplemental material for Displacement Following Minimally Invasive vs Open Lateralizing Calcaneal Osteotomy: A Direct Comparison in a Cadaveric Model Supplemental material, sj-pdf-1-fao-10.1177_24730114261463248 for Displacement Following Minimally Invasive vs Open Lateralizing Calcaneal Osteotomy: A Direct Comparison in a Cadaveric Model by Hirbod Abootalebi, William Mayer, Erin Bigney, Oreoluwa Alugo, Rowan Urquhart and Jacob Matz in Foot & Ankle Orthopaedics
